# Synergistic Combinations of FDA-Approved Drugs with Ceftobiprole against Methicillin-Resistant Staphylococcus aureus

**DOI:** 10.1128/spectrum.03726-22

**Published:** 2022-12-15

**Authors:** Amar Deep Sharma, William G. Gutheil

**Affiliations:** a Division of Pharmacology and Pharmaceutical Sciences, School of Pharmacy, University of Missouri—Kansas City, Kansas City, Missouri, USA; Keck School of Medicine of the University of Southern California

**Keywords:** library screening, drug repurposing, *Staphylococcus aureus*, synergy, antibiotic drug resistance, methicillin-resistant *Staphylococcus aureus*, ceftobiprole, ceftaroline, cloxacillin

## Abstract

New strategies are urgently needed to address the public health threat of antimicrobial resistance. Synergistic agent combinations provide one possible pathway toward addressing this need and are also of fundamental mechanistic interest. Effective methods for comprehensively identifying synergistic agent combinations are required for such efforts. In this study, an FDA-approved drug library was screened against methicillin-resistant Staphylococcus aureus (MRSA) (ATCC 43300) in the absence and presence of sub-MIC levels of ceftobiprole, a PBP2a-targeted anti-MRSA β-lactam. This screening identified numerous potential synergistic agent combinations, which were then confirmed and characterized for synergy using checkerboard analyses. The initial group of synergistic agents (sum of the minimum fractional inhibitory concentration ∑FIC_min_ ≤0.5) were all β-lactamase-resistant β-lactams (cloxacillin, dicloxacillin, flucloxacillin, oxacillin, nafcillin, and cefotaxime). Cloxacillin—the agent with the greatest synergy with ceftobiprole—is also highly synergistic with ceftaroline, another PBP2a-targeted β-lactam. Further follow-up studies revealed a range of ceftobiprole synergies with other β-lactams, including with imipenem, meropenem, piperacillin, tazobactam, and cefoxitin. Interestingly, given that essentially all other ceftobiprole-β-lactam combinations showed synergy, ceftaroline and ceftobiprole showed no synergy. Modest to no synergy (0.5 < ∑FIC_min_ ≤ 1.0) was observed for several non-β-lactam agents, including vancomycin, daptomycin, balofloxacin, and floxuridine. Mupirocin had antagonistic activity with ceftobiprole. Flucloxacillin appeared particularly promising, with both a low intrinsic MIC and good synergy with ceftobiprole. That so many β-lactam combinations with ceftobiprole show synergy suggests that β-lactam combinations can generally increase β-lactam effectiveness and may also be useful in reducing resistance emergence and spread in MRSA.

**IMPORTANCE** Antimicrobial resistance represents a serious threat to public health. Antibacterial agent combinations provide a potential approach to combating this problem, and synergistic agent combinations—in which each agent enhances the antimicrobial activity of the other—are particularly valuable in this regard. Ceftobiprole is a late-generation β-lactam antibiotic developed for MRSA infections. Resistance has emerged to ceftobiprole, jeopardizing this agent’s effectiveness. To identify synergistic agent combinations with ceftobiprole, an FDA-approved drug library was screened for potential synergistic combinations with ceftobiprole. This screening and follow-up studies identified numerous β-lactams with ceftobiprole synergy.

## INTRODUCTION

Pathogenic bacteria are becoming increasingly drug resistant, with some now virtually untreatable (1–4). There has concurrently been a lack of new antibacterial agents to counter this threat (5–8). Methicillin-resistant Staphylococcus aureus (MRSA) is one of the ESKAPE (Enterococcus faecium, Staphylococcus aureus, Klebsiella pneumoniae, Acinetobacter baumannii, Pseudomonas aeruginosa, and Enterobacter species) pathogens and a common cause of both nosocomial and community-acquired infections (9–12). It is characterized by resistance to most commonly used β-lactam antibiotics and to many other antibiotic classes and agents (13). High-level resistance to β-lactam antibiotics in MRSA is due to its acquisition of a novel β-lactam-resistant penicillin-binding protein (PBP), PBP2a (14, 15). PBP2a has substantially reduced affinity for classical β-lactamase-resistant β-lactams, such as methicillin (14, 16), due to a restricted active site (17). While MRSA has remained susceptible to vancomycin, resistance to vancomycin in MRSA is slowly increasing (18). Relatively recently, two new cephalosporin β-lactam antibiotics have been developed which are active against MRSA due to their ability to inhibit PBP2a: ceftobiprole (19, 20) and ceftaroline (21, 22). Resistance to ceftobiprole and ceftaroline has, however, also emerged (23–27).

There is a well-recognized need for new agents and approaches to combat the growing problem of antimicrobial resistance (3, 7). Drug combination-based approaches offer the prospect of improving treatment efficacy and reducing the emergence of resistance to currently effective agents (28–32). Synergistic and antagonistic agent combinations are also of high value as probes of bacterial physiology (28, 33, 34). Methods for identifying synergistic agent combinations range from testing one to several antibiotic combinations for synergy to the screening of substantial chemical compound libraries (28, 33, 35, 36).

In a prior study (37), an FDA library screen was performed against MRSA for agents which could act synergistically with cefoxitin—a β-lactam antibiotic to which MRSA is highly but not completely resistant. This screen identified several FDA-approved agents that act synergistically with cefoxitin, including the interesting finding of strong synergy with floxuridine. However, none of the cefoxitin synergistic agents could return cefoxitin’s MIC to a therapeutic level. In this study, a similar screen was performed of an FDA-approved drug library against MRSA in the absence and presence of ceftobiprole—a newer β-lactam designed to circumvent PBP2a-based β-lactam resistance. The goal of this effort was to comprehensively identify FDA-approved drugs that can act synergistically with ceftobiprole. These results are interesting from a mechanistic perspective and suggest several ceftobiprole combinations which may have clinical potential.

## RESULTS AND DISCUSSION

This study used the same FDA-approved drug library, target MRSA strain (ATCC 43300; F-182), and screening approach previously reported for a −/+cefoxitin screen (37). Library screening was performed using 25 μM of each library compound, one set in the absence of ceftobiprole and one set in the presence of sub-MIC (0.25 μg mL^−1^ = 1/8×MIC) ceftobiprole. Any FDA compound which showed activity in the absence or presence of ceftobiprole was added to a merged hit list, and MICs were determined for the agents on this list in both the absence and presence of 0.25 μg mL^−1^ ceftobiprole. The MICs for compounds with a minimum MIC of ≤3.1 μM under any tested condition (−/+ceftobiprole) are listed in Table 1. The degree of the effect of the added ceftobiprole on the test agent MIC is indicated by the L2_(−/+ceftobiprole)_ values:

(1) $${\text{L2}}_{\left(-/+\text{ceftobiprole}\right)}={\text{ log}}_{\text{2}}\left({\text{MIC}}_{-\text{ceftobiprole}}/{\text{MIC}}_{+\text{ceftobiprole}}\right)$$

**TABLE 1:** FDA library anti-MRSA hit MICs sorted by the greatest apparent decrease in MICs with the addition of 0.25 μg mL^−1^ ceftobiprole

Compound	MIC (μM)		Min_MIC (μM)	L2_(–/+BPR)_^*b*^
	–BPR^*a*^	+BPR		
Cloxacillin	25	1.6	1.6	4
Gatifloxacin	0.78	9.8E–2	9.8E–2	3
Gemcitabine	2.4E–2	3.1E–3	3.1E–3	3
Daptomycin	0.39	9.8E–2	9.8E–2	2
Doxycycline	0.78	0.20	0.20	2
Floxuridine	2.4E–2	6.1E–3	6.1E–3	2
Methacycline	1.6	0.39	0.39	2
Moxifloxacin	0.20	4.9E–2	4.9E–2	2
Sitafloxacin	4.9E–2	1.2E–2	1.2E–2	2
Tetracycline	3.1	0.78	0.78	2
Vancomycin	0.78	0.20	0.20	2
Ciprofloxacin	0.39	0.20	0.20	1
Difloxacin	0.78	0.39	0.39	1
Doxifluridine	1.6	0.78	0.78	1
Levofloxacin	0.39	0.20	0.20	1
Marbofloxacin	3.1	1.6	1.6	1
Nadifloxacin	9.8E–2	4.9E–2	4.9E–2	1
Novobiocin	9.8E–2	4.9E–2	4.9E–2	1
Pefloxacin	1.6	0.78	0.78	1
Rifapentine	3.1	1.6	1.6	1
Sparfloxacin	1.6	0.78	0.78	1
Balofloxacin	2.4E–2	1.2E–2	1.2E–2	1
5-Fluorouracil	3.1	3.1	3.1	0
Retapamulin	4.9E–2	4.9E–2	4.9E–2	0
Rifabutin	9.8E–2	9.8E–2	9.8E–2	0
Rifampin	1.2E–2	1.2E–2	1.2E–2	0
Valnemulin	4.9E–2	4.9E–2	4.9E–2	0
Rifaximin	4.9E–2	4.9E–2	4.9E–2	0
Mupirocin	0.39	3.1	0.39	−3

a BPR, ceftobiprole.

b ${\text{L2}}_{\left(-/+\text{BPR}\right)}={\text{log}}_{2}\left(\frac{{\text{MIC}}_{-\text{BPR}}}{{\text{MIC}}_{+\text{BPR}}}\right)$.

**FIG 1:**
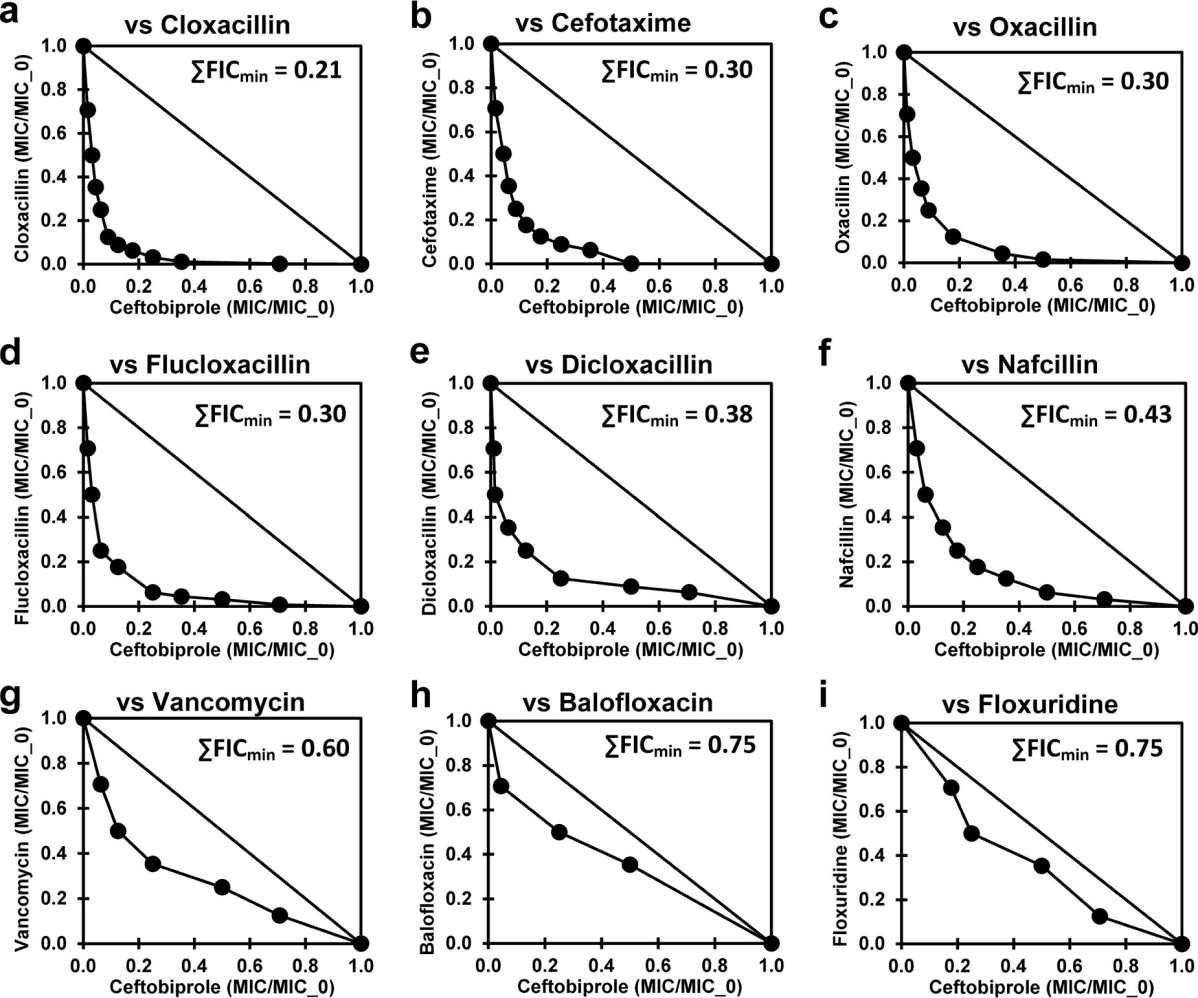
Checkerboard assay results for combinations of ceftobiprole with the best synergistic agents listed in the top section of Table 2. Isobolograms for combinations of ceftobiprole (*x* axes) with (*y* axes) cloxacillin (a), cefotaxime (b), oxacillin(c), flucloxacillin (d), nafcillin (e), dicloxacillin (f), vancomycin (g), balofloxacin (h), and floxuridine (i). ∑FIC_min_ values are shown in the insets.

Checkerboard assays were then used to confirm and characterize prospective ceftobiprole synergistic agents (L2_(−/+ceftobiprole)_ ≥ 2) and for the one prospective ceftobiprole antagonistic agent (mupirocin; L2_(−/+ceftobiprole)_ ≤ 2). Cloxacillin demonstrated particularly strong synergy with ceftobiprole (sum of the minimum fractional inhibitory concentration (∑FIC_min_ = 0.21) (Fig. 1a), and several other β-lactamase-resistant β-lactams not in the original library were then added to the checkerboard analysis (cefotaxime, ceftaroline, oxacillin, nafcillin, cloxacillin, dicloxacillin, and flucloxacillin) to evaluate the scope of this synergy (Fig. 1; Table 2). The most synergistic agent combinations for ceftobiprole are with β-lactamase-resistant β-lactams. Cloxacillin was the most synergistic of these, followed by cefotaxime, oxacillin, flucloxacillin, dicloxacillin, and nafcillin (Fig. 1a to f). Dicloxacillin and flucloxacillin also had relatively low intrinsic (without ceftobiprole) MICs (2.2 μM or 1.0 μg mL^−1^ each) (Table 2), consistent with prior observations for dicloxacillin against MRSA (38).

**TABLE 2:** Checkerboard assay results for potentially synergistic and antagonistic agents^*a*^

Reference	Agent	MIC (μM)	MIC (μg/mL)	∑FIC_min_^*b*^	∑FIC_max_^*c*^
Table 1; Fig. 1	Cloxacillin	35	15	0.21	1
	Cefotaxime	71	32	0.30	1
	Oxacillin	71	28	0.30	1
	Flucloxacillin	2.2	1.0	0.30	1
	Dicloxacillin	2.2	1.0	0.38	1
	Nafcillin	71	29	0.43	1
	Vancomycin	0.78	1.1	0.60	1
	Balofloxacin	0.02	6.7E–3	0.75	1
	Floxuridine	0.024	5.9E–3	0.75	1
	Rifaximin	0.049	3.9E–2	0.80	1
	Daptomycin	0.28	0.45	0.83	1.1
	Gemcitabine	0.024	6.3E–3	0.96	1.1
	Tetracycline	8.8	3.9	1	1.2
	Doxycycline	0.28	0.12	1	1.2
	Moxifloxacin	0.14	5.6E–2	1	1.2
	Sitafloxacin	0.035	1.4E–2	1	1.2
	Gatifloxacin	0.39	0.15	1	1.4
	Rifabutin	0.086	7.3E–2	1	1.4
	Mupirocin	0.78	0.39	1	2.7
Fig. 2	Imipenem	17	5.3	0.26	1
	Meropenem	31	12	0.38	1
	Ceftaroline fosamil	8.8	6.6	1	1.2
	Cefoxitin sodium	55	25	0.48	1
	Tazobactam	500	150	0.385	1
	Piperacillin Sodium	35	19	0.43	1
	Tazobactam/piperacillin (1:1 wt:wt)	58:32	18:18	0.50	1
Fig. 2e	Cloxacillin	0.54	0.23^*d*^	0.50	1

a The upper section lists agents identified in Table 1 and several additional β-lactamase-resistant β-lactams (Fig. 1) against MRSA (ATCC 43300). The middle section lists additional follow-up agents (Fig. 2) against MRSA. The bottom row shows the results of a test of the best MRSA synergistic agent (cloxacillin) against an MSSA strain (ATCC 25923) (Fig. 2e). The MIC for ceftobiprole was 2 μg mL^−1^.

b Lower ∑FIC_min_ values indicate greater degrees of synergy, with values closer to 1 indicative of agent additivity (no synergy).

c Higher ∑FIC_max_ values indicate greater degrees of antagonism, with values closer to 1 indicative of additivity (no antagonism).

d MIC versus ceftobiprole = 0.25 μg mL^−1^.

Vancomycin demonstrated only weak synergy with ceftobiprole (Fig. 1g; ∑FIC_min_ = 0.6). Ceftobiprole-vancomycin synergy has previously been observed (39), and several clinical studies of β-lactam combinations with vancomycin have been reported and recently reviewed (40). Floxuridine, which demonstrated strong synergy with cefoxitin in a prior study (37), demonstrated effectively no synergy with ceftobiprole (Fig. 1i). Daptomycin also showed effectively no synergy with ceftobiprole in this study (Table 2) but has demonstrated synergy in prior studies (41, 42). Mupirocin demonstrated significant antagonism (∑FIC_max_ = 2.7; ∑FIC_min_ = 1.0). Mupirocin is a topical antibiotic used for skin infections and to clear MRSA from nasal passages (43) that targets isoleucine tRNA synthetase (44). Antagonism of β-lactam activity in S. aureus via modulation of the stringent stress response by mutations or mupirocin has been observed previously (45–47).

Following these observations, several additional potentially synergistic β-lactams were investigated (Table 2; Fig. 2). Among these, imipenem showed good synergy with ceftobiprole (Fig. 2a, ∑FIC_min_ = 0.26). Ceftobiprole showed no synergy with ceftaroline, its PBP2a-inhibiting cognate (Fig. 2c), perhaps indicative of similar essential PBP selectivity patterns (20, 22). Cefoxitin, which has a much different PBP selectivity pattern (PBP4 selective) than imipenem or cloxacillin (PBP1 selective) (48–50), also demonstrated modest synergy with ceftobiprole. Cloxacillin demonstrated significant but substantially less synergy with ceftobiprole against a methicillin-sensitive S. aureus (MSSA) strain (Table 2, bottom; Fig. 2e). This observation indicates that some of the observed synergy between cloxacillin and ceftobiprole in MRSA is likely due to ceftobiprole interactions with PBPs other than PBP2a. A combination of ceftobiprole with piperacillin-tazobactam has also been reported to show synergy (51). Modest MIC synergy was observed in this study for ceftobiprole with tazobactam and piperacillin, singly and in combination (Table 2; Fig. 2g to i). The isobologram of ceftobiprole with piperacillin was noticeably asymmetric, with low piperacillin concentrations substantially enhancing the ceftobiprole activity (Fig. 2g). A similar asymmetry was also apparent with imipenem (Fig. 2a) and cloxacillin (Fig. 1a.), whereas dicloxacillin seemed to show a reversed asymmetry (Fig. 1e).

**FIG 2:**
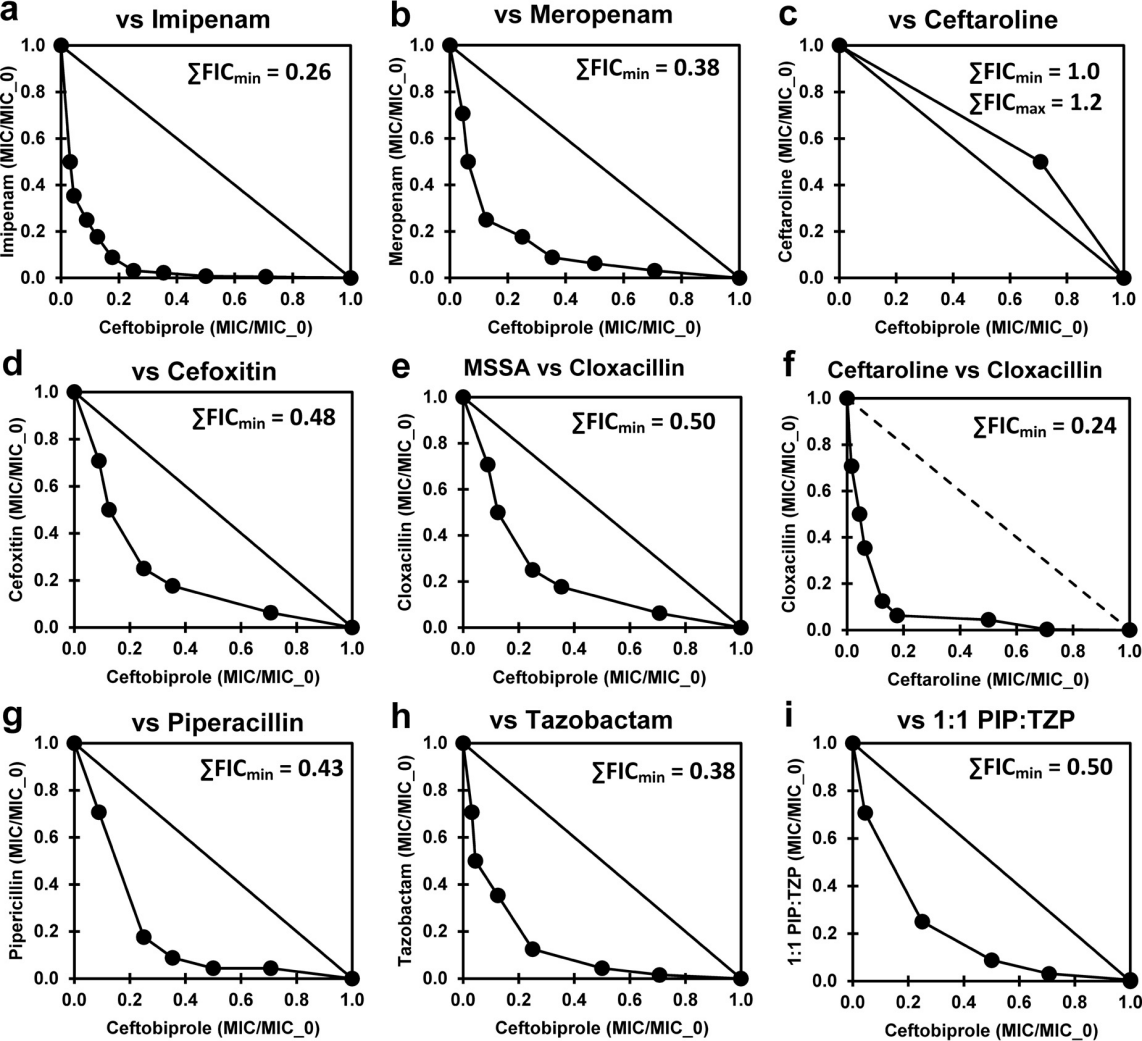
Checkerboard assay results for combinations of ceftobiprole with the additional synergistic agents and MSSA listed in the middle and bottom sections of Table 2. Isobolograms for combinations of ceftobiprole versus imipenem (a), meropenem (b), ceftaroline (c), cefoxitin (d), cloxacillin in MSSA (e), cloxacillin (f), piperacillin (g), tazobactam (h), and a 1:1 (wt/wt) piperacillin (PIP)-tazobactam (TZP) mixture (i). ∑FIC_min_ values are shown in the insets.

It is somewhat tempting to interpret these ceftobiprole synergies in terms of which PBP or PBPs must be targeted to account for the observed effects. However, such a conclusion cannot be reliably deduced from the present information, given that these β-lactams (i) have substantially different selectivities (20, 22, 48, 49, 52, 53); (ii) can all effectively inhibit more than one PBP in S. aureus; and (iii) generally have high intrinsic MICs against MRSA (Table 2), meaning that each agent at their respective MICs will effectively inhibit multiple PBPs. Additional study of the molecular basis of these synergies is required. From a clinical perspective, the good synergies with and low intrinsic MICs of flucloxacillin and dicloxacillin demonstrate that effective combinations of β-lactams with ceftobiprole are possible.

## MATERIALS AND METHODS

### General.

The FDA-approved drug library of 978 compounds was obtained from Selleckchem. Sterile 384-well microtiter plates were purchased from Corning (catalog number 3680), 96-well U-bottom polypropylene storage plates were acquired from Becton, Dickinson (catalog number 351190), and sterile 96-well microtiter plates were obtained from MidSci (catalog number TP92096). Other reagents were purchased from standard sources and were reagent grade or better. The main bacterial strain used in this study was methicillin-resistant Staphylococcus aureus (MRSA) strain F-182 (ATCC 43300). The methicillin-sensitive S. aureus (MSSA) strain used in this study was Seattle 1945 (ATCC 25923).

### MRSA versus FDA −/+ceftobiprole library screen.

The FDA-approved drug library was delivered in columns 1 to 11 in 96-deep well plates, 12 plates total, with each sample well containing 100 μL of a 10-mM solution of a compound dissolved in dimethyl sulfoxide (DMSO). A diluted working library at 100 μM in 96-well plates was prepared by serial dilution in DMSO as described previously (37). Two sets of library screening plates were then prepared for the −ceftobiprole and +ceftobiprole screens by transferring 5 μL from the working library plates into the wells of 384-well plates (3 plates in each of two sets) using a Biomek 3000 liquid handling workstation. The plates were frozen at −80°C and dried under a strong vacuum (<50 μmHg) in a Genevac Quattro centrifugal concentrator.

To each well in each set was added 20 μL cation-adjusted Mueller-Hinton (CAMH) broth containing 4,000 CFU of MRSA (ATCC 43300) and either no ceftobiprole for the −ceftobiprole screens or 0.25 μg mL^−1^ ceftobiprole (equal to ~1/8× MIC) for the +ceftobiprole screens. These additions were performed using an Integra Viaflo Assist automated multichannel pipettor in a Labconco biosafety level 2 (BSL-2) cabinet. FDA-approved drug library compounds were present at 25 μM in these two plate sets (−ceftobiprole and +ceftobiprole). The plates were incubated for 48 h at 35°C. Fresh CAMH broth (10 μL) was added to the wells of these four sets of plates, followed by incubation for 2 h at 35°C to restart active cell growth. To the wells of these plates was then added 6 μL of 100 μg mL^−1^ resazurin (sodium salt) (54–56). The plates were incubated for another 2 h at 35°C, and the fluorescence excitation at 570 nm and emission at 600 nm (Ex_570_/Em_600_) (Promega Technical Bulletin TB317) of the wells was measured using a Molecular Devices SpectraMax M5 multimode microplate reader. Library screening data were processed and analyzed using Matlab scripts (MathWorks). Based on the values for known active and inactive antibacterial agent controls, a cutoff value between the active and inactive compounds was selected, and lists of active wells in each screening set (−ceftobiprole, +ceftobiprole) were generated. These lists were merged to give a pooled hit list, as described previously (37).

### MIC determination.

MICs were determined by hit picking 5-μL samples from the working library plates (100 μM) into the first columns of 384-well plates (two sets, one set for the −ceftobiprole MICs and one for +ceftobiprole MICs). These samples were then serially diluted in steps of two across the plates with DMSO using an Integra Viaflo Assist automated multichannel pipettor. The last column was left blank (DMSO only). These plates were frozen at −80°C and dried under a strong vacuum as described above. To each well in each set was added 20 μL cation-adjusted Mueller-Hinton (CAMH) broth containing 4,000 CFU of MRSA (ATCC 43300) and either no ceftobiprole for the –ceftobiprole MICs or 0.25 μg mL^−1^ ceftobiprole for the +ceftobiprole MICs (the MIC for ceftobiprole was 2 μg mL^−1^). This provided the MIC plates with 12.5 μM as the highest FDA-tested agent concentration. The plates were incubated for 48 h at 35°C. Fresh CAMH broth (10 μL) was added to the wells of these four sets of plates, followed by incubation for 2 h at 35°C to restart active cell growth. To the wells of these plates was then added 6 μL of 100 μg mL^−1^ resazurin. The plates were incubated for another 2 h at 35°C, and the Ex_570_/Em_600_ fluorescence of the wells was measured as described above. The MICs were determined using a cutoff midway between the known active and inactive samples. All MICs were determined at least in triplicate to ensure reproducibility. The results from these MIC determinations are shown in Table 1.

### Checkerboard assays to confirm synergy or antagonism with ceftobiprole.

Several agents demonstrated apparent synergistic activity with ceftobiprole (L2 values of ≥2 in Table 1). Checkerboard assays to confirm synergy (57, 58) were performed using individually purchased compounds in 384-well plates using serial dilution steps of √2 (1.41) in CAMH, starting with a ceftobiprole concentration of 4 μg mL^−1^ (2× MIC) diluted in the top to bottom dimension and a concentration of the FDA agent starting at 2× to 8× MIC diluted in the left to right dimension. The last row (row 16) and column (column 23) in each dimension had a zero concentration of the respective agent. The last column (column 24) was used as a blank control with no ceftobiprole or FDA test agent. After the dilutions were completed, each well contained ceftobiprole and FDA test agents in 10 μL medium at 2× the experimental concentration. To each well in these plates was then added 10 μL CAMH broth containing 4,000 CFU MRSA, which diluted these agents to 1× their experimental concentrations, and the plates were incubated for 24 h at 35°C. Fresh CAMH broth (10 μL) was then added to each well; the plates were incubated at 35°C for 2 h to restart the bacterial metabolism, 6 μL of 100 μg mL^−1^ resazurin was added, and the plates were incubated for an additional 2 h. The Ex_570_/Em_600_ fluorescence of the wells was measured as described above. All checkerboard assays were performed at least in triplicate and the data averaged. Checkerboards were also performed using serial dilutions in DMSO to ensure consistency with the dilutions performed in CAMH, but the dilutions in CAMH gave more precise (less variable) results, likely due to the fewer plate manipulations required for the CAMH dilution experiments.

### ∑FIC_min_ and ∑FIC_max_ calculations.

Fractional inhibitory values were calculated using the following formula (58–60):

(2) $$\frac{{\text{MIC}}_{\text{A}/\text{B}}}{{\text{MIC}}_{\text{A}/0}}\;+\;\frac{{\text{MIC}}_{\text{B}/\text{A}}}{{\text{MIC}}_{\text{B}/0}}={\text{FIC}}_{\text{A}}+{\text{FIC}}_{\text{B}}=\sum^{\;}\text{FIC}$$

MIC_A/0_ is the MIC of A alone, and MIC_B/0_ is the MIC of B alone. For each data point in an isobologram, MIC_A/B_ is the MIC of A in the presence of some (MIC) concentration of B, and MIC_B/A_ is the MIC of B in the presence of some (MIC) concentration of A. FIC_A_ (the fractional inhibitory concentration of A) corresponds to the fractional MIC for A, and FIC_B_ corresponds to the fractional MIC for B. The sum of these values for each individual data point is the ∑FIC, which is at a minimum near the origin of the isobologram. The minimum ∑FIC value over all the isobologram data points is the ∑FIC_min_. Similarly, the highest value over all the isobologram data points is the ∑FIC_max_ (Table 2). ∑FIC_min_ values of ≤0.5 are indicative of synergy, ∑FIC_max_ values of ≥4 are indicative of antagonism, and values between 0.5 and 4 are indicative of additive effects (57).
